# Supplementary material for: Uncovering the Professional Landscape of Clinical Research Nursing: A Scoping Review with Data Mining Approach
Source: Nurs Rep. 2025 Jul 24;15(8):266. doi: 10.3390/nursrep15080266 (PMC12388699; doi:10.3390/nursrep15080266)
Supplement: Supplementary file 1 [file nursrep-15-00266-s001.zip › nursrep-3746308-supplementary/Supplementary File (S1).pdf]

**Supplementary File S1.** Detailed search strategy

| | |
|---|---|
| MEDLINE: 1336 Accessed: December 2024 | ((((((((clinical research, nursing[MeSH Terms])) OR (clinical research nurse*[Title/Abstract])) OR (research nurse*[Title/Abstract])) OR (clinical trial nurse*[Title/Abstract])) OR (research nurse coordinator*[Title/Abstract])) AND ((((((((scope of practice[MeSH Terms]) OR (nurse's role[MeSH Terms])) OR (professional competence[MeSH Terms])) OR (clinical competence[MeSH Terms])) OR (competenc*[Text Word])) OR (role[Text Word])) OR (barrier*[Text Word])) OR (professional identity[Text Word])) OR (responsibilit*[Text Word])) |
| CINAHL: 6307 Accessed: December 2024 | ((MH "Clinical Research Nurses") OR TI clinical research nurse* OR AB clinical research nurse* OR TI research nurse* OR AB research nurse* OR TI clinical trial nurse* OR AB clinical trial nurse* OR TI research nurse coordinator* OR AB research nurse coordinator*) AND ((MH "Scope of Practice") OR (MM "Nursing Role") OR MH professional competence OR MH clinical competence OR TX competenc* OR TX role OR TX barrier* OR TX professional identity OR TX responsibilit*)) |
| PsycInfo: 1764 Accessed: December 2024 | ((MH "Clinical Research Nurses") OR TI clinical research nurse* OR AB clinical research nurse* OR TI research nurse* OR AB research nurse* OR TI clinical trial nurse* OR AB clinical trial nurse* OR TI research nurse coordinator* OR AB research nurse coordinator*) AND ((MH "Scope of Practice") OR (MM "Nursing Role") OR MH professional competence OR MH clinical competence OR TX competenc* OR TX role OR TX barrier* OR TX professional identity OR TX responsibilit*)) |
| Web of Science: 1225 Accessed: December 2024 | (((((((("clinical research, nursing")) OR ("clinical research nurse*")) OR ("research nurse*")) OR ("clinical trial nurse*")) OR ("research nurse coordinator*")) AND (((((((("scope of practice") OR ("nurse's role")) OR ("professional competence")) OR ("clinical competence")) OR (competenc*)) OR (role)) OR (barrier*)) OR ("professional identity")) OR (responsibilit*))))) |
| ProQuest Dissertation and Theses | ((clinical research nurse) OR (clinical research nursing) OR (research nurse) OR (clinical trial nurse) OR (research nurse coordinator)) AND ((scope of practice) OR (nurse role) OR (professional competence) OR (clinical competence) OR (role) OR (barrier) OR (professional identity) OR (responsibility)) |
