# Supplementary material for: Uncovering the Professional Landscape of Clinical Research Nursing: A Scoping Review with Data Mining Approach
Source: Nurs Rep. 2025 Jul 24;15(8):266. doi: 10.3390/nursrep15080266 (PMC12388699; doi:10.3390/nursrep15080266)
Supplement: Supplementary file 1 [file nursrep-15-00266-s001.zip › nursrep-3746308-supplementary/Supplementary File (S3).pdf]

Supplementary File (S3). Data mining procedures and validation steps.

To systematically explore the textual data extracted from the included studies, a data mining workflow was developed using R. The analysis was conducted through a combination of natural language processing and topic modeling techniques to identify latent thematic structures within the corpus of study findings.

S.3.1 Data preparation

First, the 'Findings' sections of the included studies were imported from a CSV file and pre-processed to prepare the textual data for analysis. Text cleaning was performed using the 'tm' and 'tidytext' packages, involving several key steps: converting all text to lowercase, removing punctuation and numbers, eliminating standard English stopwords, and applying a set of customized stopwords (e.g. "findings", "results", "also", and "across") to reduce noise in the dataset. After stripping whitespace and finalizing the pre-processing, a Document-Term Matrix (DTM) was created to represent the frequency of terms across the corpus. Following data cleaning, documents with zero word counts were excluded to ensure robust analysis, and the refined corpus was used for topic modeling.

S.3.2 LDA

To identify the optimal number of topics for the LDA model, we used the 'ldatuning' package, which evaluates topic coherence and model fit across different solutions. The tuning procedure tested models with a range of 2 to 10 topics, using the established metrics. The tuning results can bee seen in Figure S1. which supported the selection of the most coherent and interpretable number of topics for the dataset.

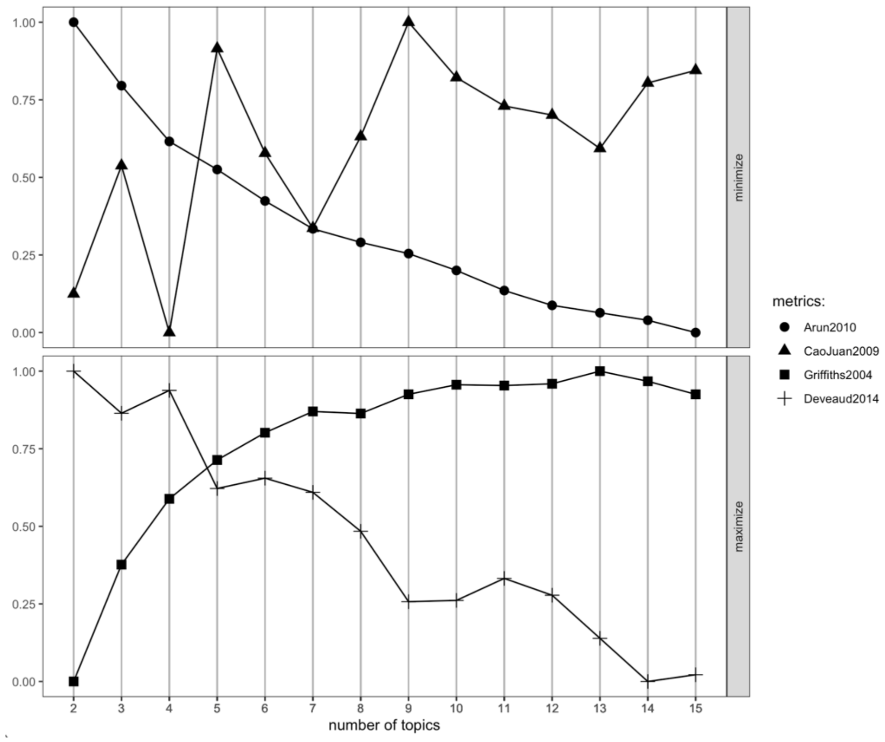

Figure S1. Optimal number of topics selection

This process enabled a data-driven approach to topic identification, reducing researcher bias and ensuring that the themes extracted from the findings were grounded in the underlying structure of the corpus.

Following the construction of the DTM, LDA was applied to identify underlying themes within the findings of the included studies. Using the Gibbs sampling method and setting the number of topics to **k=4**, the LDA model successfully extracted four distinct topics, each characterized by the most representative terms and keywords. The four topics were interpreted as follows (Figure S2), based on the analysis of the ten highest-probability terms within each topic:

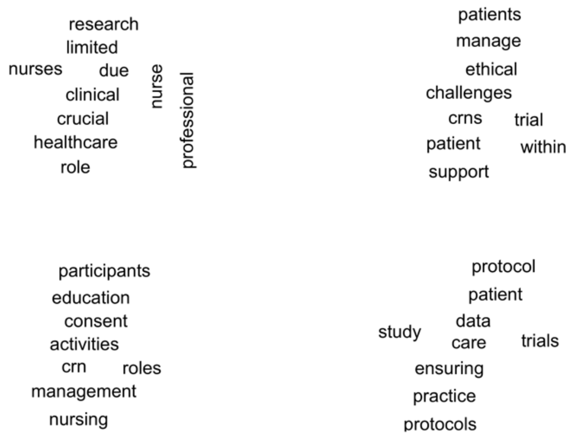

Figure S2. Word clouds for each topic (topic one at the top left corner)

Each document was assigned to the topic with the highest posterior probability. The distribution of documents across the four topics was relatively balanced, with 39 studies (30.7%) assigned to Topic 4, 32 studies (25.2%) to Topic 3, and 28 studies (22.0%) each to Topics 1 and 2. This distribution suggests that the included literature tends to concentrate around two primary areas: (1) the operational aspects of clinical research nursing, including protocol management and training (Topics 3 and 4), and (2) the professional identity and challenges of CRNs within contexts (Topics 1 and 2). Posterior probabilities from the LDA model indicated a moderate level of topic mixture within documents, with no extreme dominance of a single topic across the entire corpus.

S.3.3 Correlation matrix

To explore the relationships between the topics identified through the LDA model, a correlation matrix was computed based on the topic probability distributions across the documents. The analysis revealed generally low to moderate negative correlations between the topics. Specifically: Topic 1 (Role perception and team integration) showed a moderate negative correlation with Topic 2 (Barriers) (r = -0.47) and weaker negative correlations with Topic 3 (Professional competencies and development) (r = -0.18) and Topic 4 (Clinical trial management) (r = -0.06). Topic 2 also exhibited moderate negative correlations with Topic 3 (r = -0.37) and Topic 4 (r = -0.42), indicating a degree of divergence between discussions of ethical challenges and those addressing practical training and care delivery. Topic 3 and Topic 4 were negatively correlated (r = -0.44), suggesting that studies focusing on educational and managerial aspects tend to be distinct from those centered on patient care and protocol execution.

S.3.4 MCA

To further explore the relationships between categorical variables and the topics identified through the LDA model, a Multiple Correspondence Analysis (MCA) was conducted. This method allows the visualization of associations between qualitative variables and supports the identification of patterns across the dataset. The analysis was performed using the FactoMineR and factoextra packages in R. The MCA was applied to a subset of variables from the included studies, specifically *Year*, *Country*, *Design*, and *Topic*. All variables were transformed into categorical factors to meet the requirements of the MCA procedure. The analysis generated two-dimensional coordinates for each study, enabling the graphical representation of documents in a reduced dimensional space based on their shared attributes (Figure 3 in the manuscript). To enhance the interpretability of the results, individual studies were plotted on the MCA map and colored according to their assigned topic. Ellipses were added around each topic group to illustrate the dispersion and potential clustering of studies within the MCA space. Variable contributions to the dimensions were also assessed to evaluate which categories of *Year*, *Country*, and *Design* were most influential in defining the principal axes of the analysis. Finally, to improve the readability of the graph, labels for individual studies were selectively displayed based on their contribution (cos^2) to the overall solution, using differentiated thresholds to prioritize the most representative cases.

S.3.5 Clustering

Following the MCA, a clustering analysis was conducted to identify potential groupings of studies based on their MCA-derived coordinates. Specifically, a K-means clustering algorithm was applied using the kmeans function in R. To determine the optimal number of clusters, a scree plot of the within-cluster sum of squares (WCSS) was generated, allowing for the identification of the "elbow point" that best balanced model complexity with explanatory power (Figure S3).

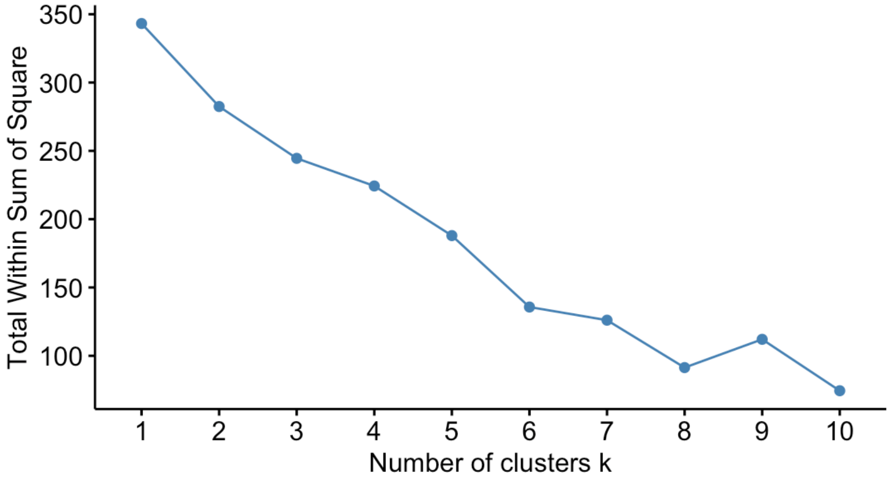

Figure S3. Elbow criterion

Once the optimal number of clusters was established (k=4), K-means clustering was performed, and each study was assigned to a cluster accordingly. The distribution of LDA-derived topics within each cluster was then examined to assess the alignment between the thematic structures identified through topic modeling and the document groupings derived from clustering. This comparison revealed a high degree of consistency between the clusters and the previously defined topics, supporting the robustness of the LDA results and providing an additional layer of validation for the thematic distinctions observed across the corpus (Figure 4 in Result

section). In order do gain deep comprehension of the hidden topics the k-means dendogram was computed identifying substantial 7 sub-clusters (Figure S4).

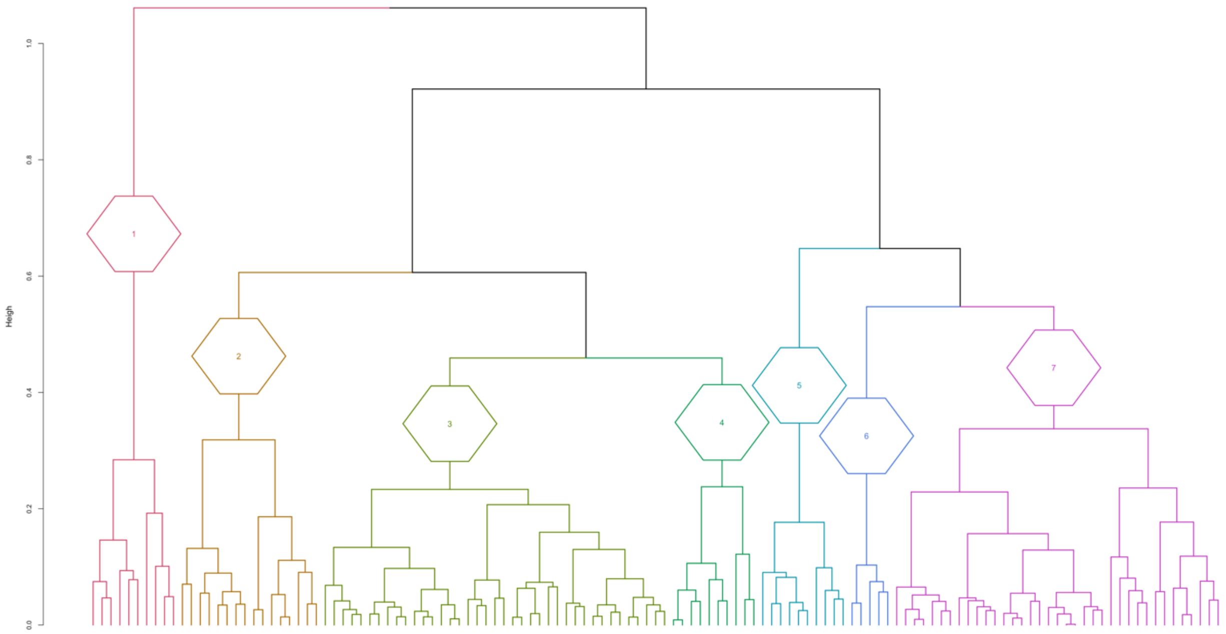

Figure S4. K-means dendogram
